# A rare nonsynonymous variant in the lipid metabolic gene HELZ2 related to primary biliary cirrhosis in Chinese Han

**DOI:** 10.1186/s13223-016-0120-6

**Published:** 2016-04-04

**Authors:** Ping Li, Guanting Lu, Li Wang, Ying Cui, Ziyan Wu, Si Chen, Jing Li, Xiaoting Wen, Haoze Zhang, Shijie Mu, Fengchun Zhang, Yongzhe Li

**Affiliations:** Key Laboratory of Rheumatology and Clinical Immunology, Department of Rheumatology and Clinical Immunology, Peking Union Medical College Hospital, Peking Union Medical College and Chinese Academy of Medical Sciences, Ministry of Education, Beijing, China; Department of Blood Transfusion, Tangdu Hospital, The Fourth Military Medical University, Xi’an, China

**Keywords:** Primary biliary cirrhosis, HELZ2, Replication study, Lipid metabollism

## Abstract

**Background:**

Several genome-wide association studies of primary biliary cirrhosis (PBC) in European and Japanese origins have shown significant association of dozens of genetic loci contributive to the susceptibility of PBC. Most of the loci were related to immune response pathway. In this study, we tested whether the lipid metabolic gene HELZ2 was associated with the pathogenesis of PBC.

**Methods:**

In 586 PBC cases (358 in case 1 group and 201 in case 2 group) and 726 healthy controls of Chinese Han, six nonsynonymous SNPs were genotyped by MassArray iPLEX. The same control were used for the two groups of PBC cases. Allele frequencies were calculated by χ^2^ test based on 2 × 2 contingency tables. All data were analyzed using the PLINK tool set. The odds ratio (OR) and 95 % confidence interval (95 % CI) were calculated, and *p* values (corrected for multiple testing by Bonferroni adjustment) less than 0.05 were considered statistically significant.

**Results:**

The A allele of rs79267778 was significantly associated with PBC (OR_combined_ = 4.204 [1.670–10.582], *p*_*combined*_ = 1.87E−04). It changed the amino acid at position 1904 (NM_001037335) from Threonine (ACG) to Methionine (ATG). This site was highly conserved in mammals and predicted to be POSSIBLY DAMAGING with a score of 0.469 by PolyPhen-2. It’s further predicted that T1904 M could INCREASE the protein stability with a confidence at 25.18 % under the condition of pH 7.0 and 37 °C.

**Conclusion:**

The result was the first time to show evidence of the lipid metabolic gene HELZ2 related to autoimmune disease, at least in PBC of Chinese Han.

## Background

Primary biliary cirrhosis (PBC) is a common autoimmune liver disease, characteristically associated with anti-mitochondrial antibodies and affecting up to 1 in 1000 women over 40 years of age [1]. Though GWAS studies have found dozens of gene associated with PBC in European and Japanese populations. The genetic factors contributing to the pathogenesis still remain elusive.

HELZ2 [OMIM 611265], also known as PRIC285 or PDIP1, is a 2649 amino acid nuclear helicase protein, being a part of the peroxisome proliferator activated receptor α interacting (PRIC) complex [2]. It functions as a nuclear transcriptional coactivator for PPARA and PPARG, as well as other nuclear receptors (RXRA, THRA, THRB) [2, 3]. Inferred from items of gene ontology, this gene is mainly related with cellular lipid metabolic process [4] and liver regeneration [5].

It had been reported that HELZ2’s binding partner, Pparg could regulate the expression of inflammatory genes such as Ccl3, Ccl7, Cxcl10 and Tgtp in mice [6]. This gene also played an important role in innate immune responses [7] and related to immune-related diseases, such as HCV/HIV infection [8], osteoarthritis [9], acne vulgaris [10]. As to itself, HELZ2 possessed the ability to bind proteins important for immune responses, such as BCL6 [11] and ISG15 [12]. Therefore, we inferred that HELZ2 might be associated with autoimmune diseases. Since it could affect liver regeneration, this gene had the promising potentiality to contribute to the pathogenesis of PBC.

In order to verify the assumption, we selected 6 nonsynonymous SNPs in the coding region of HELZ2 from 1000 Genomes Project for Asian populations with MAF >2 % and genotyped in 586 PBC cases and 726 healthy controls with Chinese Han. Of the 6 SNPs, the T allele of rs79267778 was found to significantly associated with PBC (OR_combined_ = 4.204 [1.670−10.582], *p*_*combined*_ = 1.87E−04). It changed the amino acid at position 1904 (NM_001037335) from Threonine (ACG) to Methionine (ATG). Conservation analysis showed this site was highly conserved in mammals and might influence the function of HELZ2. The result was the first time to show evidence of the lipid metabolic gene HELZ2 related to autoimmune disease, at least in PBC of Chinese Han.

## Methods

### Information of the recruited subjects

The peripheral blood mononuclear cells (PBMCs) of a total of 385 PBC subjects (case 1 group) and 726 age-matched healthy controls were collected by the Rheumatology Department of Peking Union Medical College Hospital and 201 PBC samples (case 2 group) collected from multiple medical centers in China (Table 1). The healthy control were used for the two groups of cases. All patients fulfilled the criteria of the American Association for the Study of Liver Diseases for primary biliary cirrhosis [13]. All patients and controls were unrelated individuals of Han Chinese ethnicity by self-report. The study was approved by the ethics committee of the Peking Union Medical College hospital and all subjects gave informed consent.

**Table 1:** Samples information

Groups	Total	Genders		Ages		
		Female	Male	Minimum	Maximum	Mean ± SD
Case 1	385	358	27	16	75	34.69 ± 12.61
Case 2	201	185	16	18	80	35.53 ± 9.87
Control	726	680	46	11	79	34.20 ± 11.25

### Genotyping of selected SNPs in HELZ2

The genomic DNA of each sample was extracted using QIAamp DNA mini kit (Qiagen, German), and SNP genotyping was performed by the MassArray iPLEX system (Sequenom, USA) at Beijing DnaLead Co. LTD. All procedures were performed according to the manufacturer’s instructions. Approximately 10 ng of genomic DNA was amplified by multiplex PCR and the amplicons subjected to locus-specific single-base extension reactions. The extended products were desalted and transferred to a 384-element SpectroCHIP array. Allele detection was performed using MALDI-TOF mass spectrometry, and the mass spectrograms were analyzed by the MassArray TYPER software v4.0 (Sequenom, USA). 6 SNPs in the coding sequence of HELZ2 were selected for genotyping in the 2 cohorts of Chinese Han (385 cases/726 controls and 201 cases/726 controls) by MassArray iPLEX.

### Association analysis of the genotyped SNPs

The genotyped SNPs were tested for Hardy–Weinberg equilibrium (HWE) in the patient and control populations, and any SNPs that deviated from HWE (p < 0.05 in the control group) were excluded from subsequent analyses. Allele frequencies were calculated by χ^2^ test based on 2 × 2 contingency tables. Since the incidence of PBC was both gender and age related, we took gender and age as covariates in our analysis. All data were analyzed using the PLINK tool set. The odds ratio (OR) and 95 % confidence interval (95 % CI) were calculated, and *p* values (corrected for multiple testing by Bonferroni adjustment) less than 0.05 were considered statistically significant.

## Results

### Structural survey of the genomic region with HELZ2

**Fig. 1:**
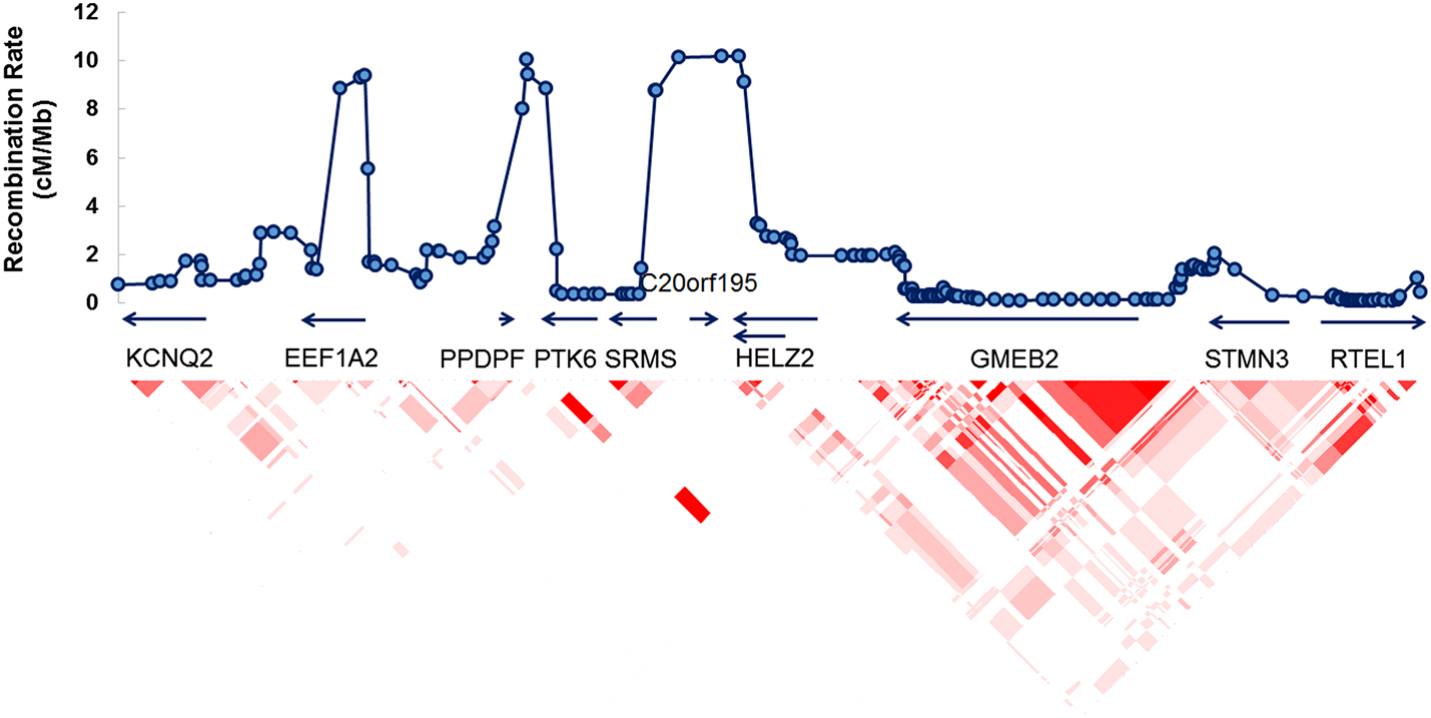
Linkage disequilibrium of the region containing HELZ2. *Upper panel* showed the recombination rate (cM/Mb) (hg19, estimated using all populations of HapMap Phase 2: *CEU* Utah residents of Northern and Western European ancestry, *JPT* Japanese in Tokyo, Japan, *CHB* Han Chinese in Beijing, China, *YRI* Yoruba in Ibadan, Nigeria. Genetic recombination rates were represented by *grey line*, and genes within the region were shown in the *middle panel*. The *lower panel* showed the pairwise linkage disequilibrium between SNPs in this region. The color of each *diamond* represented the pairwise linkage between SNPs (measured as r^2^) defined by the *upper left* and the *upper right* *sides* of the *diamond*. Shading represented the magnitude of pairwise LD, with a *red-to-white* gradient reflecting higher to lower LD values

We extracted structural variation data of the region (chr20:62,089,439-62,305,592) from the Database of Genomic Variants [14] and mapped against human reference genome (hg19). HELZ2 was encircled by common deletion polymorphisms. Besides, HELZ2 was located in a region with high recombination rate (cM/Mb) (Fig. 1, upper panel). The LD pattern showed that HELZ2 was in a weakly linked block (Fig. 1 lower panel). Based on these preliminary information, 6 nonsynonymous SNPs in the coding region of HELZ2 were selected from 1000 Genomes Project for Asian populations with MAF >2 % for further genotyping (Table 2).

**Table 2:** Association of SNPs with primary biliary cirrhosis in Chinese Han

SNP ID	Risk allele	Cohort 1			Cohort 2			Odds ratio [95 % CI] (combined)	*p* value (combined)
		Case 1^a^	Control^b^	*p* value	Case 2^a^	Control^b^	*p* value		
rs3810481	G	0.407	0.462	0.054	0.434	0.462	0.086	0.801 [0.647–0.991]	0.044
rs3810483	G	0.125	0.170	0.210	0.154	0.170	0.340	0.822 [0.599–1.127]	0.23
rs79267778	T	0.024	0.006	3.62E−03	0.019	0.006	2.07E−02	4.204 [1.670–10.582]	1.87E−04
rs3810486	A	0.393	0.418	0.174	0.405	0.418	0.333	0.820 [1.416–2.347]	0.3107
rs6089924	A	0.004	0.011	0.057	0.009	0.011	0.405	0.377 [0.100–1.426]	0.2248
rs114867526	A	–	–	–	–	–	–	–	–

^a^Two groups of PBC cases

^b^The same control group

### Association of rs79267778 (T1904 M) with PBC in Chinese Han

**Fig. 2:**
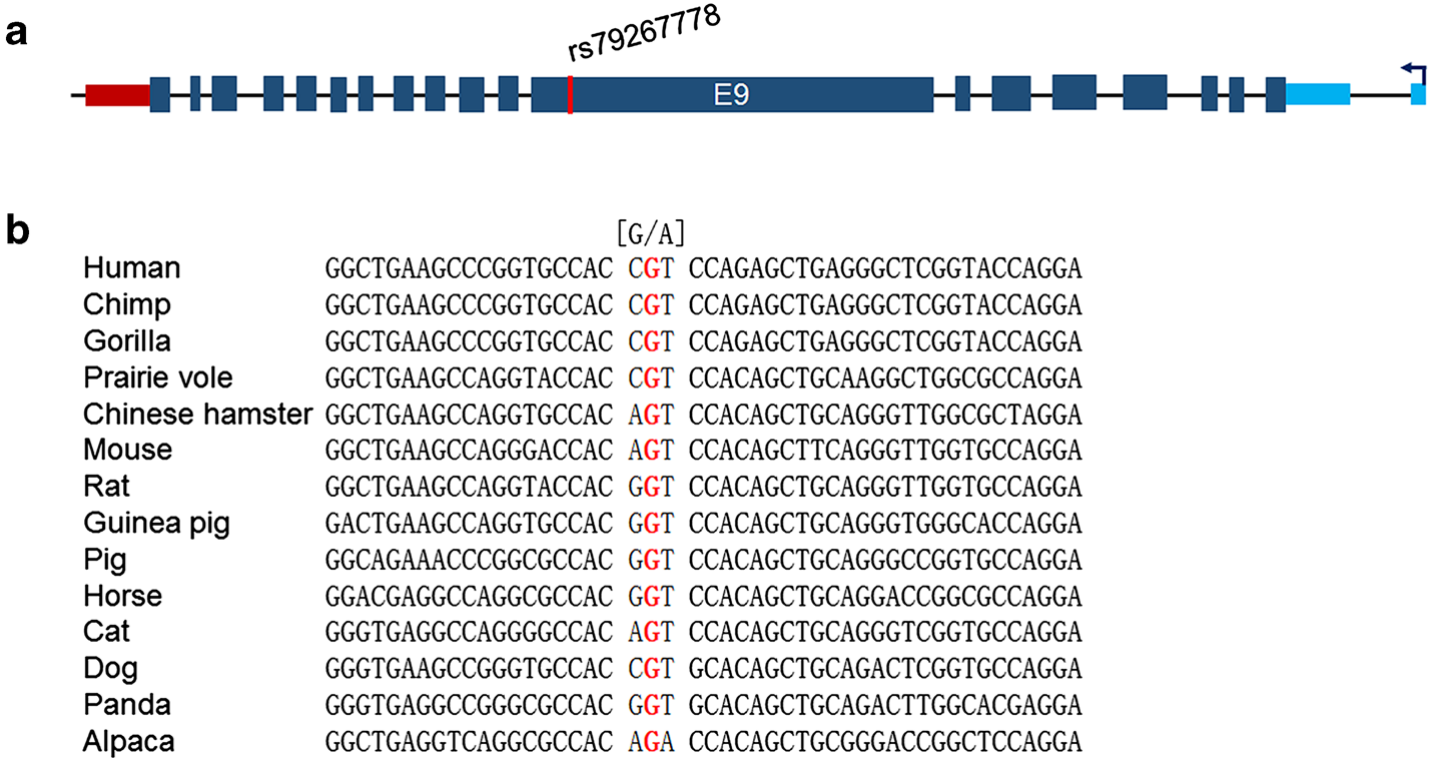
Diagram of HELZ2 and conservation of rs79267778. **a** The structure of HELZ2 gene. *Green* and *red* boxes represented 5′ UTR and 3′ UTR, respectively. *Blue box* indicated exons, *thin line* between *boxes* represented introns, *red line* indicated the location of rs114867526. *Arrow* indicated the direction of transcription. **b** The alignment of sequences around rs79267778 from 14 species

Of the 6 nonsynonymous SNPs, five were successfully genotyped and one (rs114867526) failed. After analysis, only one SNP, rs79267778, was found to be significantly associated with PBC in the first cohort of PBC samples (358 cases/726 controls) (*p* = 3.62E−03). In order to validate the associatioin, 201 PBC samples from multiple medical centers were genotyped and compared with same control samples, rs79267778 was also marginally associated with the disease (*p* = 2.07E−02). After combination analysis, the SNP was strongerly linked with the pathognesis of PBC (OR_combined_ = 4.204 [1.670–10.582], *p*_*combined*_ = 1.87E−04) (Table 2). rs79267778 located in the largest exon (E9) of HELZ2 (Fig. 2a). The risk allele (A allele) changed the amino acid at position 1904 (NP_001032412) from Threonine (ACG) to Methionine (ATG).

### T1904 M was highly conserved in mammals

Sequences around rs79267778 of 14 species were extract and aligned by the molecular evolutionary genetics analysis software, MEGA v4.0 [15]. The first (A) and second (C) nucleotide of the codon for Threonine (ACG) was highly conserved, with the third varied greatly (Fig. 2b). This mutation was predicted to be POSSIBLY DAMAGING with a score of 0.469 by PolyPhen-2. The web-based software, MuStab (Predicting Mutant Protein Stability Change) was applied to predict if the SNP could influence the stability of HELZ2. Under the condition of pH 7.0 and 37 °C, T1904 M could INCREASE the protein stability with a confidence at 25.18 %.

## Discussion

Previous GWAS had identified dozens of loci susceptible to primary biliary cirrhosis in European and Japanese population, such as HLA-DQB1, IL12A, IL12RB2, STAT4, SPIB, IRF5, IKZF3, IL7R, CD80, TNFSF15 and so on [16–19]. The majority of these genes participated in immune responses. Recently, the pivotal gene for energy metabolism, PPARG, has been linked with some kinds of immune-related diseases, such as HCV/HIV infection, osteoarthritis and acne vulgaris. As the important coactivator for PPARG, HELZ2 could bind proteins important for immune responses, such as BCL6 and ISG15. Therefore, we speculated that HELZ2 might be associated with autoimmune diseases. Since it could affect liver regeneration, HELZ2 had the highly potentiality to contribute to the pathogenesis of primary biliary cirrhosis.

Using a panel of 6 nonsynonymous SNPs of HELZ2 selected for association in 2 cohorts of Chinese Han, our results showed significant association of a rare SNP, rs79267778 with PBC in Han Chinese (OR_combined_ = 4.204 [1.670−10.582], *p*_*combined*_ = 1.87E−04).

Recently, Fairfax B. reported that a known autoimmune disease locus, rs11171739 at 12q13.2 could trans-regulate the expression of PRIC285 (a well-known name for HELZ2) in B cells and suggested peroxisome proliferator-activated receptor γ (PPARG) signaling might be involved in autoimmune pathogenesis [20]. In this study, a nonsynonymous SNP in the coding region of HELZ2 was identified to significantly associated with PBC.This was the first genetic analysis to associate HELZ2 with the pathogenesis of autoimmune disease, at least for primary biliary cirrhosis in Han Chinese. Except for PBC, there are at lease 2 types of other autoimmune-related liver diseases, such as autoimmune hepatitis (AIH) and primary sclerosing cholangitis (PSC). The relationship of HELZ2 with AIH and PSC should be elucidated further.

Since rs79267778 could change the coding of amino acid at position 1904 of HELZ2, we inferred that it might affect the structure of DNA binding domain or transcription factor binding domain of the protein. According to the conserved domains of HELZ2, it contains 7 domains, AAA domain (519–590), DNA2 domain (642–938), UvrD_C_2 domain (713–915), P-loop_NTPase domain (2150–2189), AAA_11 domain (2152–2399), AAA_12 domain (2406–2611). T1904 M does not locate in any functional domains of the protein. The stability change of HELZ2 was predicted using MuStab for T1904 M. interestingly, the SNP could INCREASE the protein stability with a confidence at 25.18 %. The stabilized HELZ2 could prolong the half-life of its ligand-dependent nuclear receptor transcription coactivator activity. However, precise molecular mechanism of the variant of HELZ2 with primary biliary cirrhosis should be elucidated further.

## Conclusion

This was the first evidence to show that the lipid metabolism-related gene, HELZ2 was linked with autoimmune disease, at least for primary biliary cirrhosis in Chinese Han population. Further studies should be performed to reveal the impact of this SNP on the function of HELZ2.
